# Sarcoidosis Presenting as a Lung Mass in a Patient With COVID-19 Infection: A Case Report

**DOI:** 10.7759/cureus.39136

**Published:** 2023-05-17

**Authors:** Sindhu C Pokhriyal, Muhammad Nabeel Pasha, Ahmad Khan, Rosine Uwiringiyimana, Hadeeqa Idris

**Affiliations:** 1 Internal Medicine, One Brooklyn Health, New York City, USA; 2 Pulmonary and Critical Care Medicine, One Brooklyn Health, New York City, USA; 3 Pulmonology, Interfaith Medical Center, New York City, USA; 4 Medicine, American University of Antigua, New York City, USA; 5 Internal Medicine, Shifa International Hospital Islamabad, Islamabad, PAK

**Keywords:** dysregulation, immune, cancer, covid-19, mass, lung, sarcoidosis

## Abstract

Coronavirus disease 2019 (COVID-19) is a viral infection caused by severe acute respiratory syndrome coronavirus 2 (SARS-CoV-2) which is known to be associated with immune dysregulation and can cause multiorgan dysfunction. Sarcoidosis is another disease associated with increased inflammatory responses due to immune dysregulation which can also affect multiple organs. Although sarcoidosis, like COVID-19 infection, can affect virtually any organ, the lungs are the most commonly affected organs. Sarcoidosis most commonly presents as lung nodules and bilateral hilar lymphadenopathy. Rarely, multiple granulomatous lesions can coalesce and manifest as lung masses, and these often mimic lung cancer. We present a case of a 64-year-old male who presented with shortness of breath and pneumonia-like symptoms for one week and a nasopharyngeal swab for SARS-CoV-2 was positive. Workup revealed a large 6.3×4.7 cm lung mass in the right upper lobe along with enlarged bilateral lymph nodes. A CT-guided lung biopsy was done which revealed non-caseating granulomas containing epithelioid cells. Other causes of granuloma like tuberculosis and fungal infections were ruled out. The patient was managed with low-dose steroids and a follow-up CT scan done after eight months revealed complete resolution of lung mass with minimal mediastinal lymphadenopathy. This is, as far as we are aware, the first case of COVID-19 infection manifesting as a lung mass that was ultimately diagnosed as sarcoidosis.

## Introduction

Increased immunoreactivity is a hallmark of both coronavirus disease 2019 (COVID-19) infection and sarcoidosis. Additionally, very less is known about the clinical and immune interaction between COVID-19 and sarcoidosis [1]. Sarcoidosis is a multisystemic disease characterized by non-caseating epithelioid granulomas which appear as nodules formed by inflammatory cells in different organs. Lungs, eyes, and skin are the commonest sites of the disease. It is a diagnosis of exclusion. When histologic evidence of non-caseating granulomatous inflammation corroborates with the clinical-radiographic findings and other possible causes of granulomas and local responses have been reasonably ruled out, a diagnosis of sarcoidosis can be made [2]. High-resolution computed tomography (HRCT) has been increasingly important in recent years for making the diagnosis of both COVID-19 infection as well as sarcoidosis. Bilateral hilar adenopathy, peribronchial-vascular thickening, and perilymphatic infiltrative lesions are the most distinctive features seen in imaging [3]. Biopsy and histopathology are usually deferred in asymptomatic as well as minimally symptomatic patients. However, there is considerable overlap between the clinical and imaging features of COVID-19 and sarcoidosis, making diagnosis frequently challenging. Therefore, in symptomatic patients, it is imperative to establish a histopathological diagnosis of sarcoidosis to guide early treatment [4]. Rarely, the illness manifests as massive masses of confluent nodular opacities, shown on CT scans as areas of lung consolidation with air bronchograms [3]. We present one such case where due to the clinical presentation of lung mass, it was further challenging to differentiate between lung cancer and sarcoidosis in our COVID-19-infected patient. To our knowledge, this is the first case of COVID-19 infection manifesting as a sarcoid lung mass.

## Case presentation

**Figure 1:**
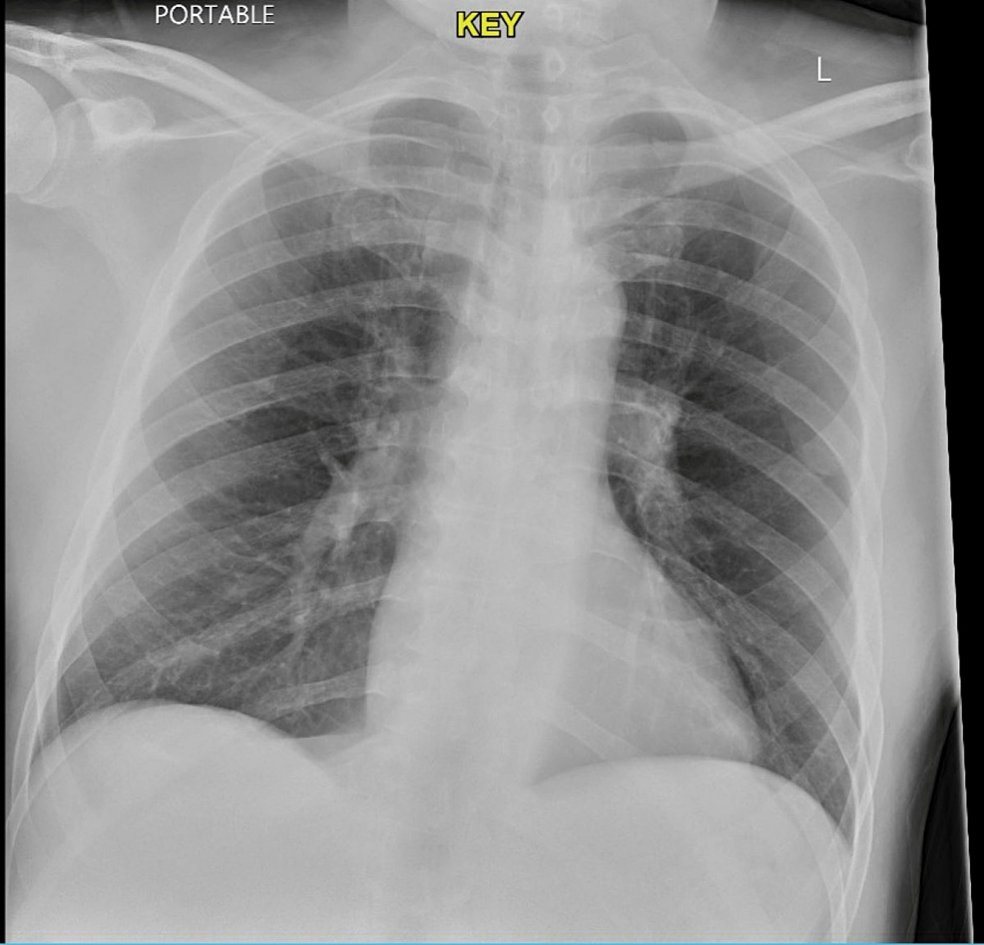
Chest x-ray at the time of admission.

A 64-year-old male with a past medical history of chronic obstructive pulmonary disease (COPD), atrial fibrillation, heart failure with reduced ejection fraction, diabetes mellitus, hypertension, stroke, and thyroid disease presented to the emergency department (ED) due to shortness of breath for one week with productive cough, chest tightness, and fatigue, not responding to home remedies and inhalers. There was no history of fever, night sweats, loss of weight, or loss of appetite. He had a past history of 25 pack years of smoking and quit smoking approximately 10 years back. His home medications included aspirin 81 mg, furosemide 40 mg, losartan 50 mg, metoprolol succinate (slow release) 100 mg, apixaban 5 mg, metformin 500 mg, albuterol 90 mcg inhaler, and symbicort 160/4.5 mcg inhaler. The patient was reported to be compliant with his medications and his inhaler technique was deemed correct. He reported receiving the COVID-19 vaccine as well as the booster dose. His peak expiratory flow rate (PEFR) at admission was 210 L/min (normal range 440-550 L/min). On examination, his vitals were stable and oxygen saturation on ambient air was 97%. Chest examination showed reduced air entry in the bilateral lower lung fields and bilateral wheezes were heard in the area of the right upper chest. The rest of the physical examination was unremarkable. A nasopharyngeal swab done for SARS-CoV-2 was positive. An x-ray chest showed bilateral lung hyperinflation (Figure 1). Of note, also is the fact that a previous x-ray chest done three months back for an episode of COPD exacerbation was normal and no interval changes were noted in the current x-ray chest.

Blood laboratory tests showed normal leukocyte count with normal differentials and were largely unremarkable (Table 1). The electrocardiogram was normal. Arterial blood gas was normal except for evidence of slight hypoxemia. pH was 7.43 (reference range: 7.35-7.45), pCO_2_ was 45 (reference range: 35-45 mmHg), pO_2_ was 69 mmHg (reference range: 80-100 mmHg), HCO_3_ was 29 (reference range: 22-26 mmol/L), and oxygen saturation was 94% (reference range: 95-99%) on room air.

**Table 1:** Patient laboratory findings at admission.

Laboratory test	Normal range	Results
White blood cell	4.5-11.0×10^3^/uL	5.2
Hemoglobin	11.0-15.0 g/dL	13.6
Hematocrit	35-46%	38
Mean corpuscular volume	80-100 fL	88.1
Platelets	130-400×10^3^/uL	388
Blood urea nitrogen	9.8-20.1 mg/dL	11
Creatinine	0.57-1.3 mg/dL	0.87
Estimated glomerular filtration rate	≥90.0 mL/min/1.73 m^2^	95
Potassium	3.5-5.1 mmol/L	4.4
Sodium	133-145 meQ/L	137
Phosphorus	2.3-4.7 mg/dL	4.9
Magnesium	1.6-2.6 mg/dL	1.8
Calcium	8.4-10.5 mg/dL	10.1
Vitamin D	30-100 ng/mL	32
D-dimer	≤500 ng/mL DDU	189
Prothrombin time	9.8-13.4 seconds	13.2
International normalized ratio	0.85-1.15 ratio	1.02
Troponin I	0.0 0.049 ng/mL	0.16, 0.17,0.16
Partial thromboplastin time	24.9-35.9 s	36
Thyroid-stimulating hormone	0.465-4.680 uIU/mL	1.9
Free thyroxine (T4)	0.78-2.19 ng/dL	1.66
Brain natriuretic peptide	10.0-100 pg/mL	299
Angiotensin-converting enzyme (ACE)	<25.0 IU/L	20.1

**Figure 2:**
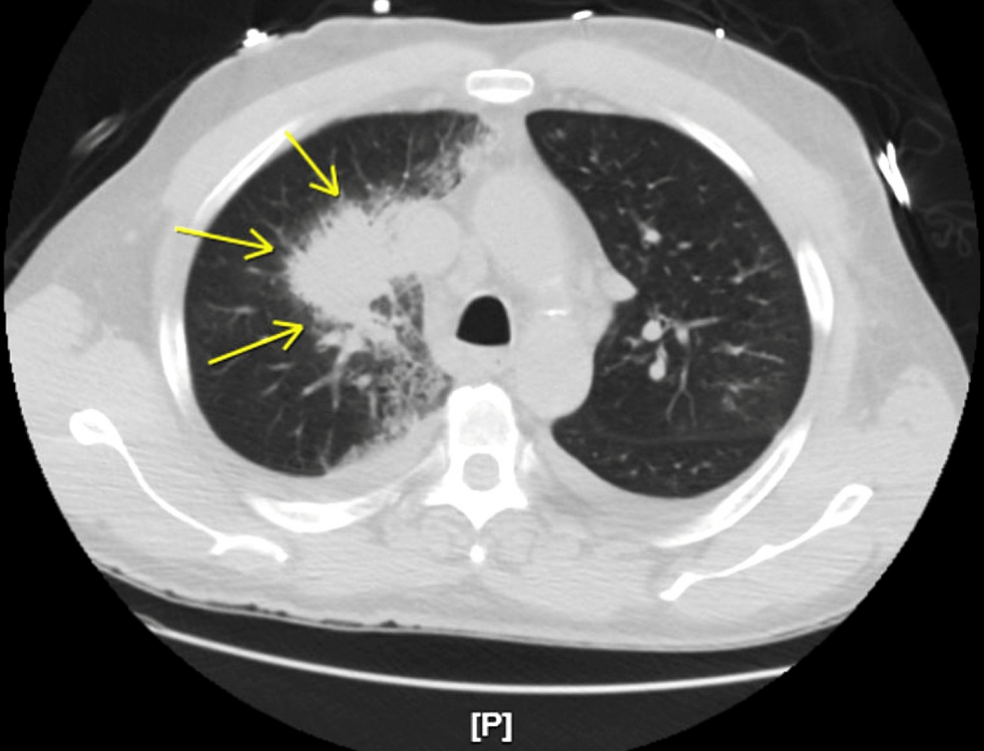
CT scan showing a lung mass sized 6.3×4.7×3.2 cm and bilateral small pleural effusion.

**Figure 3:**
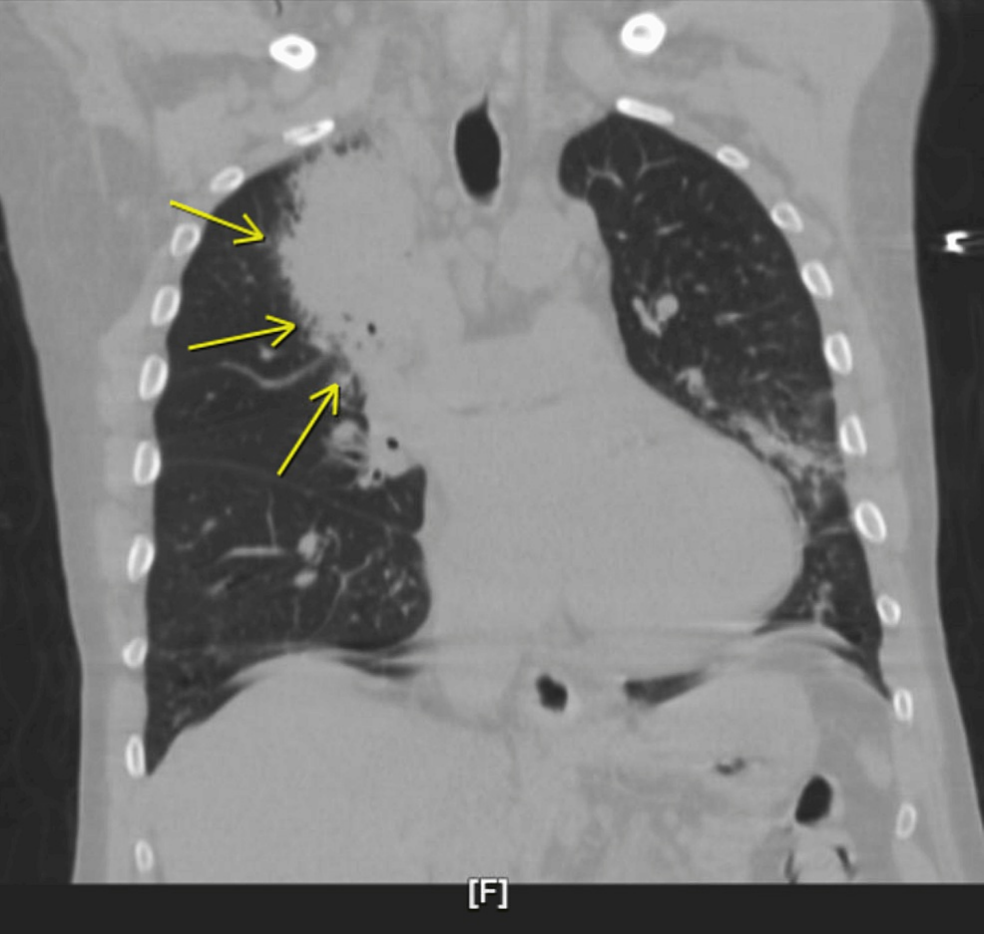
CT chest showing right lung mass.

The computed tomography (CT) chest showed a small bilateral pleural effusion and a 6.3×4.7×3.2 cm lobulated mass in the right upper lobe with a surrounding pulmonary infiltrate that was inseparable from the mediastinal side of pleura suspicious of consolidation in the region of a possible primary tumor (Figures 2, 3). There were enlarged bilateral mediastinal lymph nodes.

**Figure 4:**
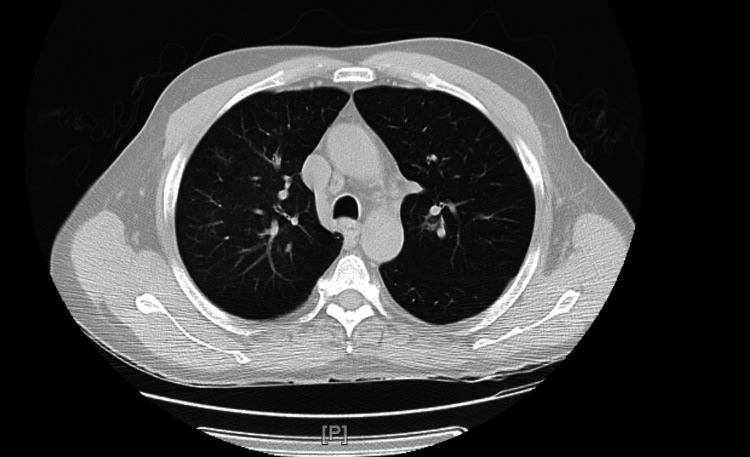
CT scan eight months after steroid therapy showed complete disappearance of the lung mass.

Acid-fast bacilli (AFB) smears were negative for tuberculosis (TB). Sputum culture showed no growth. He was admitted to the unit and managed with methylprednisolone, doxycycline, ceftriaxone, and ipratropium-albuterol nebulizations. A Quantiferon immunoassay was negative for TB. Legionella and Mycoplasma workup was negative for atypical pneumonia. CT-guided biopsy and lymph node aspiration was done. The patient showed clinical improvement with an improvement of PEFR to 290 L/min by day 3 of admission. He was discharged home with a follow-up appointment in the chest clinic. At the time of discharge, the patient was on Symbicort 160-4.5 mcg, albuterol-ipratropium inhalers, and prednisolone 40 mg daily. The patient was advised to follow-up in the chest clinic within a week of discharge. Histopathology revealed chronic inflammation with vague epitheloid non-caseating granulomas and was negative for malignancy. Other causes of granulomas, including tuberculosis and fungal infection, were also ruled out using special stains and cultures from lymph node aspirates. Angiotensin-converting enzyme (ACE) levels were sent and were on the higher side of the normal range (Table 1). The patient had improved symptomatically and the PEFR in the clinic was 360 L/min which was his baseline. The prednisolone was reduced to 10 mg per day and then gradually tapered to 4 mg per day after two weeks. A repeat CT scan done eight months later demonstrated the complete disappearance of the lung mass with a significant reduction in the mediastinal lymphadenopathy (Figure 4). The patient received a total of 10 months of prednisolone therapy and was doing well at his last two-year follow-up visit with no reported flares.

## Discussion

Sarcoidosis is a multisystemic disease that is characterized by hallmark histopathological features of non-caseating granulomas [1]. It rarely presents as a dominant nodular mass or as discrete nodules that resemble cancer or infection [5]. When an unusual presentation is seen, it is recommended that a clear diagnosis should be obtained by doing biopsies at multiple sites. Multiple biopsies have a better diagnostic yield and reduce the false negative rate of biopsies in sarcoidosis [5]. Not all patients with sarcoidosis need diagnostic procedures and treatment. Patients with asymptomatic disease and coincidental findings of bilateral hilar lymphadenopathy may be managed with surveillance alone [6]. Indications for starting treatment in sarcoidosis include poor performance status due to exhaustion, weight loss, arthralgias, and shortness of breath [7]. Our patient was symptomatic with dyspnea, cough, chest tightness, and severe fatigue. Hence, in our patient, a diagnostic procedure like a CT-guided biopsy was warranted to establish the diagnosis and guide treatment. Furthermore, it was imperative to rule out lung cancer in our patient owing to the large size of the lung mass and the clinical presentation.

Sarcoidosis remains to be known largely as a disease of unknown etiology [8]. As we proceed to highlight the immunological pathophysiology of sarcoidosis, we raise the subject of whether or not the pulmonary mass in our patient was caused by a sarcoid-like reaction following infection with the SARS-CoV-2 pathogen. Sarcoid-like reactions are autoimmune responses mimicking the one seen in sarcoidosis. This reaction can be triggered by different etiologies, the most common being infections. Malignancy, medications, and other autoimmune reactions have also been implicated in sarcoid-like reactions [8,9]. These triggers act like foreign antigens that activate antigen-presenting cells, such as interstitial dendritic cells and alveolar macrophages. They then cause T and B lymphocyte recruitment and increase in activity leading to an interferon γ (IFN-γ) mediated hyperimmune state in the body [6]. Capaccione et al. in their review suggested that these common processes and mechanisms might help explain how SARS-CoV-2 infection can perhaps trigger pulmonary sarcoidosis-like clinical presentation [6]. Behbahani et al. presented the case of a 72-year-old female who developed a sarcoid granuloma after a SARS-CoV-2 infection. In this case, the patient presented with erythema nodosum and panniculitis which are well-known skin manifestations of sarcoidosis [10]. Ramstein et al. in their study explained that when compared to control patients, sarcoid lungs have a 60% higher chance of an accelerated release of IFN-γ which is responsible for the formation of these non-caseating granulomas [11]. Furthermore, Wu et al., in their study, associated IL-17 with the pathogenesis of the COVID-19 cytokine storm and proposed that blocking this pathway would be a useful therapeutic approach [12]. Thus, a granuloma can present because of a delayed hypersensitivity reaction or an extreme immune response with infection being one of the most common triggers [6]. In our case, the patient's sarcoid-like hyperimmune state caused coalescing of multiple nodules leading to lung mass formation, and this hyperimmune state was likely triggered by SARS-CoV-2 infection.

Classically, patients with COVID-19 pneumonia show typical ground glass opacities on CT scans. However, it is important to note that at least 3% of the time, COVID-19 infection can show atypical symptoms like pleural effusion, cavitation, and lung nodules [13]. While the clinical presentation of squamous cell carcinoma associated with sarcoidosis is rare, studies have shown a correlation [11]. In our case, the large mass seen on the CT scan at the time of presentation, looked more like a coincidental finding and the patient did not exhibit any of the B symptoms of cancer. However, it is noteworthy that multiple medical publications have reported atypical pulmonary nodules on radiographic imaging of patients infected with COVID-19. For example, a recent case reports a concurrent solid nodule in a 37-year-old COVID-19 pneumonia patient who was also a renal transplant recipient. The nodule eventually on biopsy turned out to be an adenocarcinoma lung [14]. A similar case report in a healthy 40-year-old male also led to the diagnosis of adenocarcinoma in a patient with COVID-19 infection who presented with multiple bilateral round to elliptical peripheral ground-glass opacities initially attributed to COVID-19 pneumonia and later on biopsy confirmed to be adenocarcinoma of the lung [15]. Therefore, lung carcinoma was one of our first differentials and a CT-guided biopsy was done which fortunately ruled out lung cancer.

Our manuscript aimed to support ongoing research and literature to further understand and expand the correlation between COVID-19 and sarcoidosis. We suggest that it is imperative to carefully evaluate radiological findings to prevent misdiagnosis of pulmonary nodules and masses in the presence of COVID-19 infection. It is important to note that COVID-19 infection has been linked to both sarcoid-like immune reactions and malignancy.

## Conclusions

The presentation of sarcoidosis in a patient with COVID-19 infection suggests that there may be an association between viral infections like COVID-19 and exaggeration of the inflammatory process that leads to the formation of non-caseating granulomas as well as a lung mass in rare cases, such as ours. To confirm this association, additional research is required.
